# The Impact of Smartphone Addiction on PTSD Symptoms Among South African University Students: Resilience as a Protective Factor

**DOI:** 10.3390/healthcare13233087

**Published:** 2025-11-27

**Authors:** Anita Padmanabhanunni, Tyrone B. Pretorius

**Affiliations:** Department of Psychology, University of the Western Cape, Bellville 7530, South Africa

**Keywords:** smartphone addiction, South Africa, resilience, PTSD, moderation analysis

## Abstract

**Highlights:**

**What are the main findings?**

- Problematic smartphone use was significantly associated with higher levels of post-traumatic stress symptoms among South African university students.
- Resilience moderated this relationship, buffering the impact of smartphone addiction on PTSD symptoms and reducing psychological distress among individuals with higher resilience.
- Resilience did not significantly moderate the relationship between smartphone addiction and the re-experiencing or hyperarousal clusters, suggesting that these physiologically driven aspects of post-traumatic distress are less amenable to cog-nitive or emotional coping resources.

**What is the implication of the main findings?**

- Strengthening resilience may serve as an effective strategy to mitigate the negative mental health effects of excessive smartphone use in trauma-exposed populations.
- University-based mental health initiatives should integrate digital wellbeing and resilience-building interventions to promote healthier coping and reduce vulnerability to distress.

**Abstract:**

**Background**: Problematic smartphone use has emerged as a growing concern among young adults and has been linked to adverse mental health outcomes. However, limited research has examined how protective factors such as resilience may buffer the relationship between smartphone addiction and post-traumatic stress disorder (PTSD). **Methods**: Participants (*n* = 491, $\bar{X}$ age = 21.22 years) were students enrolled at a university in the Western Cape province of South Africa. They completed three standardized measures: the Smartphone Application-Based Addiction Scale, the Connor–Davidson Resilience Scale-10, and the Posttraumatic Stress Disorder Checklist for DSM-5. Moderation analyses were conducted using the PROCESS macro for SPSS to examine whether resilience moderated the association between smartphone addiction and post-traumatic stress symptoms. **Results**: Higher levels of smartphone addiction were significantly associated with elevated PTSD symptoms across all clusters. Resilience emerged as a significant protective factor, exerting direct effects on several PTSD symptom clusters and moderating the association between smartphone addiction and the avoidance and negative alterations in cognition and mood clusters. However, resilience did not significantly moderate the relationship between smartphone addiction and the re-experiencing or hyperarousal clusters, suggesting that these physiologically driven aspects of post-traumatic distress are less amenable to cognitive or emotional coping resources. **Conclusions**: These findings highlight resilience as a key buffer in the relationship between problematic smartphone use and trauma-related distress. While strengthening resilience may mitigate certain cognitive and affective dimensions of PTSD associated with excessive smartphone use, interventions may need to incorporate strategies to effectively address hyperarousal and intrusive re-experiencing symptoms among trauma-exposed students.

## 1. Introduction

The proliferation of smartphones has profoundly transformed how individuals communicate, learn, and engage with the world. While these devices offer significant advantages such as instant access to information, social connectivity, and convenience, they have also given rise to problematic patterns of use that may have detrimental psychological consequences [1]. In understanding the psychological mechanisms underlying smartphone use and its association with mental health outcomes, it is important to differentiate between adaptive and maladaptive coping strategies. Adaptive coping refers to strategies that effectively reduce psychological distress and promote long-term adjustment, such as problem-solving, seeking social support, or engaging in relaxation and mindfulness-based activities [2]. When it comes to the use of smartphones, adaptive coping can entail patterns of engagement that enhance communication, productivity, and well-being without causing interference in daily functioning or psychological distress [3]. In contrast, maladaptive coping involves behaviors that provide short-term relief but exacerbate distress over time, such as avoidance, denial, or excessive reliance on digital devices for emotional regulation [1,3]. Within the context of smartphone use, maladaptive coping may manifest as compulsive checking, scrolling, or gaming to distract from negative emotions or traumatic memories [3].

The current study focuses on smartphone addiction, which is characterized by excessive, uncontrolled, and compulsive use of smartphones, resulting in behavioral and psychological problems [4]. It is associated with symptoms of withdrawal and significant distress when access to the device is restricted. This addiction is largely fueled by excessive internet engagement, particularly through instant messaging and social media platforms that have increasingly replaced in-person interactions. Although smartphone addiction is not formally recognized as a mental health disorder, it has become a growing concern, especially among adolescents and young adults [5,6,7]. Smartphone addiction can also be differentiated from problematic smartphone use, which encompasses a broad range of behaviors reflecting excessive or poorly controlled use that interferes with daily life but may not necessarily reflect addiction [1].

Several theoretical frameworks have been proposed to explain smartphone addiction. The Compensatory Internet Use Theory (CIUT) suggests that individuals engage in excessive smartphone use as a means of escaping reality and seeking emotional gratification in the digital environment [8]. Similarly, the Integrative Pathways Model (IPM) posits that individuals with underlying psychological vulnerabilities may rely on smartphones for reassurance seeking and to maintain social connections [9]. Together, these theories highlight the psychological functions that smartphone use can serve, particularly for individuals attempting to cope with unmet emotional or relational needs.

Existing studies have highlighted the association between excessive smartphone use and adverse mental health outcomes, including stress, anxiety and depression. For instance, in their systematic review examining the prevalence of smartphone addiction and associated mental health outcomes among children and adolescents, Sohn and colleagues concluded that problematic smartphone use was significantly linked to higher levels of depression, anxiety, perceived stress, and poorer sleep quality [10]. Similar findings emerged from a systematic review of smartphone addiction and health outcomes among adults, which documented consistent associations with anxiety, depression, and even certain neurological disorders [5]. Evidence from national and population-based studies further supports these patterns. A Canadian study of youth reported that problematic smartphone use was linked to heightened anxiety, suicidal ideation, and poorer self-reported mental health [11], while a population-based study in China [12] and a national study in Singapore identified significant associations between problematic smartphone use and increased levels of depression and anxiety [13].

Perceived stress has been identified as a key antecedent of mobile phone addiction [14,15,16]. Individuals experiencing high levels of stress may turn to their phones as a way to manage or temporarily escape from negative emotions and external pressures. However, over time, this reliance on mobile phone use to regulate stress can become maladaptive, reinforcing compulsive patterns of engagement and further exacerbating emotional distress [15,16]. When considering depression and anxiety, this relationship appears to be bi-directional [7,17,18]. On one hand, excessive smartphone use, especially activities such as social media engagement and constant connectivity, can heighten stress, promote social comparison, disrupt sleep, and reduce opportunities for in-person interaction, all of which contribute to increased psychological distress [7,18]. On the other hand, individuals who already experience symptoms of anxiety or depression may be more likely to engage in problematic smartphone use as a coping mechanism to distract from negative emotions, alleviate loneliness, or seek reassurance through online interactions. Over time, this cyclical pattern can reinforce dependency and exacerbate existing mental health difficulties, creating a self-perpetuating loop between emotional distress and maladaptive technology use [7].

Although the majority of research on smartphone addiction and mental health has focused on stress, anxiety and depression, there is growing evidence of the association between problematic mobile phone use and elevated symptoms of post-traumatic stress disorder (PTSD) [19,20]. Findings suggest that individuals with elevated PTSD symptoms tend to spend more time with their smartphones, often using them as a means of avoiding or disengaging from distressing thoughts and intense emotions associated with their traumatic experiences [19,21,22]. Furthermore, recent studies have shown that all clusters of PTSD symptoms remain significantly associated with problematic smartphone use, even after accounting for the effects of depression [20].

While perceived stress has been identified as a significant antecedent of smartphone addiction, it is important to conceptually distinguish stress from PTSD. Stress represents a general psychological and physiological response to everyday challenges or pressures, such as academic workload, interpersonal conflict, or financial strain [23,24]. These stressors can vary in severity and duration and are typically transient, with individuals employing a range of adaptive coping strategies to manage them [23]. PTSD, by contrast, is a chronic and debilitating condition that arises following exposure to actual or threatened death, serious injury, or sexual violence [25]. It involves a distinct constellation of symptom clusters that persist over time and significantly impair functioning. These symptom clusters include intrusive re-experiencing (e.g., flashbacks or nightmares of the traumatic event), avoidance of reminders of the trauma, negative alterations in cognition and mood and alterations in arousal and reactivity (e.g., hypervigilance and sleep disturbances) [25].

The current study investigated the impact of smartphone addiction on symptoms of PTSD among South African university students, with a specific focus on resilience as a potential protective factor. Existing studies have confirmed that university students represent a population that is highly susceptible to adverse mental health outcomes [26,27]. The transition to university life often entails multiple stressors, including academic pressures, financial strain, social adjustment, and uncertainty about the future, all of which can increase vulnerability to psychological distress [26]. In the South African context, these challenges are compounded by socio-economic inequalities, exposure to violence and limited access to mental health resources [28]. Several studies have documented high rates of trauma exposure and PTSD among university students in the country [29,30]. For example, findings from a national student survey indicated that PTSD was among the most prevalent mental health conditions, with reported rates ranging between 21.0% and 24.5% [31]. A cross-sectional study found that the vast majority of university students (97.6%) had been exposed to at least one traumatic event [32]. The most commonly reported experiences included physical assault and transportation accidents. Gender differences were also evident in that men were more likely to report exposure to physical assault and assault involving a weapon, whereas women were more likely to report experiences of unwanted or uncomfortable sexual contact and sexual assault [32]. Other cross-sectional studies have also highlighted trauma exposure among university students and its association with adverse mental health outcomes including PTSD, alcohol use, depression and anxiety [29,33,34].

Research on mobile phone use in South Africa has primarily focused on the adoption of mobile technologies and their application in improving health-related outcomes. Studies have explored the effectiveness of mobile phone–based interventions in areas such as maternal and child health [35], medication adherence [36,37], health promotion [38] and the enhancement of primary health care services [39]. Research has also investigated the intention to adopt digital mental health interventions [40,41]. Research on smartphone addiction and its relationship with mental health outcomes in South Africa is growing. For instance, a study investigating problematic smartphone use among university students found that it was a prevalent concern, with higher levels of daily smartphone use significantly associated with greater levels of problematic use [42]. Dietrich and colleagues found that increased mobile phone use was associated with a higher risk of depression among young women [43]. Similarly, De Doncker and McLean reported a high prevalence of social media use among youth, which was associated with sleep difficulties and depression [44]. A study of children and adolescents found that extensive mobile phone use was related to elevated risk of sleep disturbances and lower health-related quality of life [45]. Another study focusing on college students reported that mobile phone and mobile gaming addiction were associated with diminished self-control [46].

Given the growing body of evidence highlighting the adverse mental health outcomes associated with problematic mobile phone use in South Africa, it is increasingly important to identify and understand the protective factors that may mitigate these risks. Resilience reflects one such protective factor and is defined as the ability to adapt positively in the face of adversity, stress, or trauma [47]. It involves dynamic processes that enable individuals to recover from challenges and maintain psychological stability. Resilient individuals typically demonstrate effective emotion regulation, cognitive flexibility, and problem-solving abilities, which buffer against the development of mental health disorders following exposure to stress or trauma [48,49]. In the context of this study, resilience is conceptualized as a protective factor that may mitigate the negative psychological effects associated with problematic smartphone use and reduce vulnerability to post-traumatic stress symptoms. Exploring such factors can provide valuable insights into how individuals maintain psychological well-being despite high levels of digital engagement and can inform the development of targeted interventions aimed at promoting healthier mobile phone use.

Based on the reviewed literature and theoretical perspectives, the following hypotheses were formulated:

**H1**: *Smartphone addiction will be positively associated with overall PTSD symptom severity and with each of the four PTSD symptom clusters.*

**H2**: *Resilience will be negatively associated with PTSD symptom severity and its clusters.*

**H3**: *Resilience will moderate the relationship between smartphone addiction and PTSD symptom severity, such that the positive association between smartphone addiction and PTSD symptom clusters will be weaker among individuals with higher levels of resilience.*

## 2. Materials and Methods

### 2.1. Participants and Procedure

Participants (*n* = 491) were students at a university in the Western Cape province of South Africa. The current paper forms part of a larger, multi-phase research project examining digital mental health, psychological well-being, and digital mental health interventions among university students. The overarching aim of the broader project is to investigate mental health challenges in young adults and to evaluate the potential of digital mental health interventions in supporting well-being. Google Forms were used to develop an electronic version of the instruments described in the Measures section. The study was undertaken between November 2023 and November 2024.

The university Registrar’s office randomly selected 1000 students and sent them an email invitation containing study information and the survey link. This process was repeated four times. A socio-demographic profile of the final sample is presented in Table 1.

As shown in Table 1, the majority of participants were women (64.8%), undergraduates (92.5%), and urban residents (64.8%). Students were drawn from all nine South African provinces, with the largest groups from the Western Cape (32.8%) and Eastern Cape (27.5%). The mean age of the sample was 21.22 years (*SD* = 3.52).

### 2.2. Measures

Participants completed a brief demographic questionnaire as well as three standardized measures: the Smartphone Application-Based Addiction Scale (SABAS) [50], the Connor-Davidson Resilience Scale-10 (CDRISC-10) [51], and the Posttraumatic Stress Disorder Checklist for DSM-5 (PCL-5) [52]. The SABAS is a 6-item instrument assessing smartphone application addiction, based on the component model of addiction (salience, mood modification, tolerance, withdrawal, conflict, relapse) [4]. Items are rated on a 6-point scale (1 = strongly disagree to 6 = strongly agree), with higher scores reflecting greater addiction. An example item is: “If I cannot use or access my smartphone when I feel like it, I feel sad, moody, or irritable.” A prior validation of the English version of the SABAS confirmed its unidimensional structure and reported α = 0.81 [50]. While there are quite a few studies in South Africa that focused on smartphone addiction, we could not find any that had used or validated the SABAS in the South African context.

The CDRISC-10 is a shortened 10-item version of the original 26-item CDRISC [53] designed to measure resilience. The items of the CDRISC-10 are scored on a 5-point scale ranging from 0 (not true at all) to 4 (true nearly all the time), and higher scores on the CDRISC-10 reflect higher levels of resilience. An example of an item of the CDRISC-10 is “having to cope with stress can make me stronger.” The study that developed the shortened CDRISC-10 found it to be a unidimensional measure of resilience and reported a reliability coefficient of 0.85 for the scores of the CDRISC-10 [51]. A validation study in South Africa used three different psychometric paradigms to confirm the unidimensionality of the CDRISC-10 and reported a reliability coefficient of 0.95 for CDRISC-10 scores in a sample of schoolteachers [54].

The PCL-5 is a 20-item measure of the presence and severity of PTSD. Items are scored on a 5-point scale ranging from 0 (not at all) to 4 (extremely), and higher scores reflect higher levels of PTSD. In addition to a total PTSD score, the PCL-5 also provides four DSM-5 symptom clusters: re-experiencing (sample item: “repeated, disturbing, and unwanted memories of the stressful experience”), avoidance (sample item: “avoiding memories, thoughts, or feelings related to the stressful experience”), negative alterations in mood and cognition (sample item: “trouble experiencing positive feelings, or example being unable to feel happiness or have loving feelings for people close to you”), and hyperarousal (sample item: “irritable behavior, angry outbursts, or acting aggressively”). The authors of the PCL-5 reported a reliability coefficient of 0.94 for PCL-5 scores and provided evidence for convergent and discriminant validity [52]. The PCL-5 has extensively been used in South Africa, and one study reported very satisfactory estimates of internal consistency (α = 0.95, ω = 0.94) for PCL-5 scores in a sample of first responders [55], while, in another study with students, reliability coefficients for the four symptom clusters ranged between 0.82 and 0.89 [56].

### 2.3. Ethics

Ethical approval to conduct the study was granted by the Biomedical Science Research Ethics Committee of the University of the Western Cape (Ethics reference: BM23/10/8, December 2023). The study was conducted according to the guidelines of the Declaration of Helsinki. Participation was voluntary, and no incentives were provided for participation.

### 2.4. Data Analysis

Responses were complete because the electronic survey required all items on a page to be answered before proceeding, leaving no missing data. IBM SPSS for Windows, version 30 (IBM Corp., Armonk, NY, USA) was used to obtain descriptive statistics (means and standard deviations), distribution indices (skewness and kurtosis), intercorrelations between study variables (Pearson’s *r*) and estimates of internal consistency (Cronbach’s alpha) for all scales. The distribution indices were used to examine the distribution of data, and data is said to be normally distributed if skewness and kurtosis values range between −2 and +2 [57]. We also examined the prevalence of PTSD in the current sample using a cutoff score of 32 [58] on the PCL-5 and the prevalence of problematic smartphone use (PSU) using a cut-off score of 23 [59].

Moderation analysis was conducted using the Hayes PROCESS macro in SPSS. In moderation analysis an interaction term consisting of the product of the independent variable and the presumed moderator (smartphone addiction X resilience) is created. A significant interaction term is indicative of a moderation effect. In this instance such a significant moderation effect would indicate that resilience interacts with smartphone addiction in impacting PTSD. Since the interaction term is a product of the other two predictors in the model, variables are mean-centered before creating the interaction term to avoid the problem of multicollinearity. The nature of the moderation effect is examined by comparing the effect of smartphone addiction on PTSD at different levels of resilience: 1 *SD* below the mean, at the mean, I *SD* above the mean. The regression lines for these three values of resilience are also plotted using the PROCESS codes for visualizing the interactions.

Prior to the moderation analysis, we examined whether age and gender were related to the dependent variable PTSD. In this regard we conducted t-tests to compare men and women, and obtained the correlation between age and PTSD. Where results were statistically significant, we added those variables (age and gender) as covariates to the moderation analysis to control for their confounding “effects.”

## 3. Results

Using a PCL-5 cutoff score of 32, it was found that slightly more than half of the sample (*n* = 257, 52.3%) reported elevated levels of PTSD. With a cut-off score of 23 on the SABAS just less than half of the sample could be classified as PSU individuals (*n* = 216, 44%). The descriptive statistics (means and standard deviations), distribution indices (skewness and kurtosis), intercorrelations between study variables, and the estimates of internal consistency (Cronbach’s alpha) are reported in Table 2.

The distribution indices (skewness and kurtosis) in Table 2 were within the acceptable range of −2 to +2 (skewness: −0.50 to 0.28; kurtosis: −1.02 to −0.05), thus indicating that the study variables were approximately normally distributed. Smartphone addiction was significantly positively associated with PTSD (*r* = 0.40, *p* < 0.001), re-experiencing (*r* = 0.39, *p* < 0.001), avoidance (*r* = 0.33, *p* < 0.001), negative alterations in mood and cognition (*r* = 0.34, *p* < 0.001), and hyperarousal (*r* = 0.36, *p* < 0.001). In all instances these associations were medium effect sizes. The positive association would indicate that higher levels of smartphone addiction are associated with higher levels of PTSD as well as higher levels of the four symptom clusters. There was no association between resilience and smartphone addiction as well as PTSD and the four symptom clusters. Table 2 also indicates that the reliability of scores produced by the various instruments was all above 0.70 (range = 0.80 to 0.94), providing strong evidence of internal consistency.

The results of the PROCESS analyses are reported in Table 3, which shows the direct effects of smartphone addiction and resilience on PTSD and the four symptom clusters. The results of one-sample t-tests showed that men and women differed significantly in terms of PTSD (women: $\bar{X}$ = 34.59, *SD* = 18.54; men: $\bar{X}$ = 28.58, *SD* = 17.87, *t* = 3.41, *p* < 0.001), re-experiencing (women: $\bar{X}$ = 8.52, *SD* = 5.65; men: $\bar{X}$ = 6.93, *SD* = 5.00, *t* = 3.16, *p* = 0.001), avoidance (women: $\bar{X}$ = 3.94, *SD* = 2.54; men: $\bar{X}$ = 3.04, *SD* = 2.28, *t* = 3.95, *p* < 0.001), negative alterations in mood and cognition (women: $\bar{X}$ = 12.24, *SD* = 7.03; men: $\bar{X}$ = 10.06, *SD* = 7.07, *t* = 3.2 to, *p* = 0.001), and hyperarousal (women: $\bar{X}$ = 9.89, *SD* = 5.84; men: $\bar{X}$ = 8.56, *SD* = 5.62, *t* = 2.39, *p* = 0.017). Age was significantly negatively associated with PTSD (*r* = −0.14, *p* = 0.001), re-experiencing (*r* = −0.11, *p* = 0. 019), avoidance (*r* = −0.13, *p* = 0.004), negative alterations in mood and cognitions (*r* = −0.15, *p* = 0.001), and hyperarousal (*r* = −0.12, *p* = 0.007). Given these relationships with the dependent variable, gender and age were added as covariates to the PROCESS analysis.

Firstly, Table 3 shows that smartphone addiction had significant direct effects on PTSD (β = 0.37, *p* < 0.001), re-experiencing (β = 0.36, *p* < 0.001), avoidance (β = 0.29, *p* < 0.001), negative alterations in mood and cognition (β = 0.31, *p* < 0.001), and hyperarousal (β = 0.34, *p* < 0.001). In the presence of resilience, the association between smartphone addiction and PTSD remains significant, which provides support for H1.

Secondly, whereas the zero-order correlations showed that resilience was not significantly related to PTSD or the symptom clusters, Table 3 shows that when resilience is considered together with smartphone addiction it had significant direct effects on PTSD (β = −0.09 *p* = 0.029), avoidance (β = −0.09 *p* = 0.041), and negative alterations in mood and cognition (β = −0.10 *p* = 0.020). This finding provides partial support for H2.

**Figure 1:**
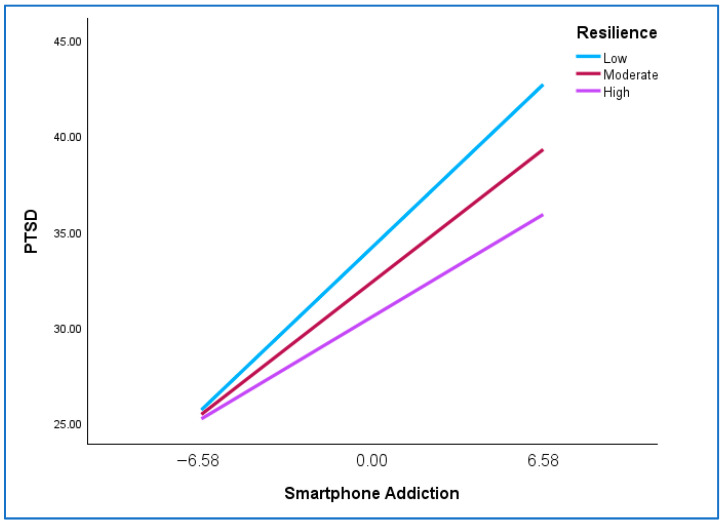
Plot of the relationship between smartphone addiction and PTSD at different values of resilience.

Lastly, Table 3 shows that the interaction term (smartphone addiction X resilience) was only significant for PTSD (β = −0.09 *p* = 0.016), avoidance (β = −0.11 *p* = 0.003), and negative alterations in mood and cognition (β = −0.09 *p* = 0.010), thus indicating a moderating role for resilience in respect of these variables which provides partial support for H3. The nature of the moderation is presented in Table 4 and illustrated in Figure 1.

Table 4 shows that, while the associations between smartphone addiction and PTSD, avoidance, and negative alterations were significant at all three levels of resilience, the standardized coefficients were consistently lower at high levels of resilience (PTSD = 0.29, avoidance = 0.18, negative alterations in mood and cognition = 0.22) than at low levels of resilience (PTSD = 0.46, avoidance = 0.40, negative alterations in mood and cognition = 0.40). In terms of effect size, the regression coefficients for the relationship between smartphone addiction and PTSD, avoidance, and negative alterations in mood and cognition at a low value of resilience would reflect medium effect sizes, while at a high value of resilience, these regression coefficients would represent small effect sizes. Figure 1 plots the relationship between smartphone addiction and PTSD at different values of resilience (Mean −1*SD*, Mean, Mean +1*SD*). As the plots for the three variables are very similar, only the plot of the relationship between smartphone addiction and PTSD is presented in the main text. The plots for the relationship between smartphone addiction and avoidance as well as negative alterations in mood and cognition are presented in Appendix A.

Figure 1 supports the results reported in Table 4 and shows that the regression line is steeper for a low value of resilience than for a high value of resilience, thus indicating that at a low level of resilience the relationship between smartphone addiction and PTSD is stronger than at a high level of resilience.

The Johnson–Neyman analysis for the relationship between smartphone addiction and PTSD found that there was no point across the observed range of resilience values where the relationship turned from significant to nonsignificant. At a value of zero for resilience, the standardized regression coefficient for this relationship was 0.66 (*p* < 0.001), while at a value of 40 for resilience, the regression coefficient was 0.23 (*p* = 0.005). In the case of avoidance, the Johnson–Neyman analysis found that at a resilience value of 37.53 the relationship between smartphone addiction and avoidance was no longer statistically significant, while for negative alterations in mood and cognition that resilience value was 38.98.

## 4. Discussion

Smartphone addiction represents a growing concern among youth, and a significant body of research has investigated its association with a range of negative outcomes including stress, depression and anxiety [6,16,21]. There has been comparatively less attention to the role of protective factors in problematic smartphone use. This study investigated the association between smartphone addiction and PTSD among South African university students, with a focus on the role of resilience as a protective factor. There were several important findings. First, smartphone addiction was significantly and positively associated with PTSD and each of its four symptom clusters namely, re-experiencing, avoidance, negative alterations in mood and cognition, and hyperarousal. This was aligned with H1 and suggests that higher levels of problematic smartphone use are linked to more severe post-traumatic stress symptoms. These findings align with existing literature suggesting that excessive smartphone use may exacerbate psychological distress by reinforcing maladaptive coping patterns such as avoidance or emotional disengagement [19,22]. Problematic smartphone use has been associated with disrupted sleep, emotion dysregulation, and rumination, all of which may heighten trauma-related symptoms [7].

These findings can be understood through the lens of the CIUT, which posits that individuals may engage in excessive smartphone use to manage or escape from distressing emotions or experiences [8]. In the context of trauma, such compensatory use may serve as an avoidant coping strategy aimed at reducing intrusive memories or emotional arousal. Similarly, the IPM suggests that individuals with underlying psychological vulnerabilities such as heightened anxiety, hyperarousal, or negative mood may rely on digital engagement for reassurance or to maintain a sense of connection. This reliance may, however, perpetuate maladaptive emotion regulation and avoidance processes, thereby maintaining or intensifying PTSD symptoms [9]. The current findings thus reinforce theoretical perspectives emphasizing the role of smartphone use as a mechanism of emotional compensation and avoidance, consistent with prior evidence that problematic smartphone use can disrupt sleep, exacerbate rumination, and undermine adaptive coping, all of which contribute to heightened trauma-related distress.

Given the association between smartphone addiction and PTSD observed in the current study, alongside established links between smartphone addiction and psychological distress in other research, it is concerning that almost half of the sample (44%) in the current study may be at risk for problematic smartphone use This proportion is somewhat lower than the rate reported in another South African study among university students, which found a 55% prevalence of PSU using a different measure of smartphone addiction [42]. Conversely, the rate observed in the current study is substantially higher than the 21.1% reported among Chinese adolescents in the study that validated a cut-off score of 23 for the SABAS [59].

Second, consistent with the existing literature, the results showed significant gender and age-related differences, with women and younger individuals reporting higher levels of PTSD respectively. Gender differences in PTSD have been attributed to women’s greater vulnerability to certain types of traumatic events that are more likely to elicit post-traumatic stress symptoms, younger age at the time of exposure, gender role socialization and barriers to mental health care [60,61]. Compared to adolescents or younger adults, increased age has been consistently associated with greater capacity for emotional regulation and enhanced cognitive re-appraisal capabilities, which could account for age-related differences in mental health outcomes [62,63].

Third, as predicted, resilience emerged as an important protective factor. Although resilience did not demonstrate significant bivariate correlations with overall PTSD scores or its symptom clusters, it exerted significant direct and moderating effects. This distinction is important to emphasize. The absence of simple correlations indicates that resilience and PTSD symptoms were not linearly related when considered in isolation. However, once other variables such as smartphone addiction were statistically controlled for, resilience significantly reduced PTSD symptom severity, particularly within the avoidance and negative alterations in mood and cognition clusters. From a theoretical standpoint, these findings are consistent with the conceptualization of resilience as a multi-modal dynamic process that fluctuates across time and circumstances and by which individuals adapt to adversity [48]. These results, which support H3, also align with prior research demonstrating the protective role of resilience in reducing vulnerability to stress-related psychopathology and promoting adaptive coping following trauma exposure [64,65,66].

The finding that resilience did not significantly moderate the relationship between smartphone addiction and the re-experiencing or hyperarousal clusters of PTSD is noteworthy. This suggests that the intrusive and physiological components of posttraumatic distress are less amenable to the buffering influence of resilience. Intrusive re-experiencing arises due to deficits in processing the traumatic event in auto-biographical memory, while hyperarousal reflects a chronic state of physiological alertness resulting from dysregulation of the autonomic nervous system [67,68]. These symptom clusters are thought to be more biologically and neurologically entrenched than the avoidance or negative cognition clusters, which are more susceptible to cognitive and emotional coping mechanisms associated with resilience [67]. This finding aligns with neurobiological models of PTSD, suggesting that heightened amygdala reactivity and impaired prefrontal regulation underlie re-experiencing and hyperarousal symptoms [68]. Consequently, even individuals with high resilience may continue to experience intrusive flashbacks or heightened physiological reactivity because these processes are rooted in automatic threat responses rather than deliberate coping efforts.

Finally, the Johnson–Neyman analyses reinforced the moderating effect of resilience. The relationship between smartphone addiction and PTSD was strongest among individuals with low resilience and gradually weakened as resilience increased. For avoidance and negative alterations in mood and cognition, there were specific points beyond which the relationship between smartphone addiction and these symptoms was no longer statistically significant. This underscores the potential threshold effects of resilience in mitigating trauma-related distress.

The findings of the study have practical implications for mental health interventions within university settings. The CIUT and IPM highlight that problematic smartphone use often arises from attempts to manage emotional distress through avoidance or reassurance-seeking, suggesting that effective interventions should target these underlying coping processes. Screening for problematic smartphone use could serve as an early indicator of vulnerability to psychological distress, particularly in trauma-exposed populations. Cognitive-behavioral interventions can subsequently help students identify and modify avoidance-based behaviour related to mobile phone use, while mindfulness-based programmes may reduce compulsive engagement by increasing awareness of emotional triggers and automatic smartphone habits [69]. The moderating role of resilience offers an important avenue for prevention and intervention. Resilience-building could be integrated into digital literacy and wellbeing programmes, promoting healthier coping strategies and more adaptive patterns of technology use. However, the finding that resilience did not moderate the association between smartphone addiction and the re-experiencing or hyperarousal clusters of PTSD means that traditional resilience-building interventions may be less effective for these symptom clusters. Instead, trauma-focused modalities such as trauma-focused cognitive behavioral therapy are more likely to facilitate the reprocessing of traumatic memories and reduce intrusive re-experiencing [70]. Universities must therefore consider a multi-layered approach that integrates individual-level interventions with broader digital wellbeing policies, psychoeducation campaigns, and culturally responsive trauma support [32].

The observed gender and age differences point to the need for targeted approaches. Female students and younger individuals may benefit from more tailored interventions addressing both trauma-related vulnerabilities and the emotional regulation difficulties that can accompany problematic smartphone use. At a policy level, universities and student support services should prioritize digital wellbeing initiatives.

Several limitations of this study should be noted. First, the cross-sectional design precludes any causal inferences regarding the directionality of the observed relationships between smartphone addiction, resilience, and post-traumatic stress symptoms. It is therefore not possible to determine whether problematic smartphone use contributes to greater trauma-related distress or whether individuals experiencing elevated PTSD symptoms are more likely to engage in maladaptive smartphone use as a coping mechanism. Longitudinal or experimental studies are needed to clarify the temporal sequence of these associations. Second, although the study employed validated self-report measures, the absence of clinician-administered diagnostic interviews means that PTSD symptoms and smartphone addiction were not formally diagnosed. As with all survey-based research, self-reported data may not fully capture clinical complexity and are subject to biases such as social desirability and recall inaccuracies. Future research could benefit from incorporating multi-method assessments, including clinical interviews or objective indicators of smartphone use. Third, the study sample was drawn from a single university, which may limit the generalizability of the findings to other student populations or to youth in different socio-economic or cultural contexts. Further research using more diverse and representative samples is recommended. Finally, although resilience was found to buffer the relationship between smartphone addiction and PTSD, the measure of resilience used in this study does not capture situational or context-specific aspects that may influence coping following trauma. Future studies should consider multidimensional measures of resilience to obtain a more nuanced understanding of its protective role.

## 5. Conclusions

This study contributes to a growing body of literature linking problematic smartphone use to adverse mental health outcomes by demonstrating a significant association between smartphone addiction and post-traumatic stress symptoms among South African university students. Importantly, it identifies resilience as a moderating factor capable of attenuating, though not eliminating, this association. The findings reinforce the conceptualization of resilience as a dynamic protective resource that can mitigate vulnerability to distress in the context of pervasive digital engagement.

## Figures and Tables

**Table 1:** Description of the sample.

Variable	Categories	$n/\bar{X}$	%/*SD*
Gender	Women	318	64.8%
	Men	163	33.2%
	Other	10	2%
Graduate Status	Undergraduate	454	92.5%
	Postgraduate	37	7.5%
Home Province	Western Cape	161	32.8%
	Eastern Cape	135	27.5%
	Gauteng	72	14.7%
	Kwazulu-Natal	41	8.4%
	Mpumalanga	32	6.5%
	Limpopo	23	4.7%
	Free State	14	2.9%
	North West	7	1.4%
	Northern Cape	6	1.2%
Residential Area	Rural	173	35.2%
	Urban	318	64.8%
Age		21.22 years	3.52

**Table 2:** Intercorrelations, descriptive statistics, distribution indices, and reliabilities.

Variable	1	2	3	4	5	6	7
1. Smartphone addiction	—						
2. Resilience	−0.02	—					
3. PTSD	0.40 **	−0.09	—				
4. Re-experiencing	0.38 **	−0.08	0.87**	—			
5. Avoidance	0.33 **	−0.08	0.80 **	0.71 **	—		
6. Negative alterations	0.34 **	−0.09 *	0.93 **	0.69 **	0.68 **	—	
7. Hyperarousal	0.36 **	0.05	0.90 **	0.67 **	0.61 **	0.79 **	—
Mean	21.92	25.19	32.57	7.99	3.63	11.52	9.43
*SD*	6.64	8.28	18.62	5.50	2.49	7.18	5.81
Skewness	−0.18	−0.52	0.12	0.28	0.14	0.18	0.21
Kurtosis	−0.57	−0.01	−0.61	−0.86	−1.03	−0.70	−0.63
Alpha	0.81	0.90	0.94	0.89	0.81	0.88	0.83

* *p* < 0.05, ** *p* < 0.001.

**Table 3:** Direct and moderating effects obtained with PROCESS analysis.

Effects	B	SE	95% CI		β	*p*
			LL	UL		
Direct Effects						
Smartphone addiction → PTSD	1.04	0.12	0.81	1.27	0.37 **	<0.001
Smartphone addiction → Re-experiencing	0.30	0.04	0.23	0.37	0.36 **	<0.001
Smartphone addiction → Avoidance	0.11	0.02	0.08	0.14	0.29 **	<0.001
Smartphone addiction → Negative alterations	0.34	0.05	0.25	0.43	0.31 **	<0.001
Smartphone addiction → Hyperarousal	0.29	0.04	0.22	0.37	0.34 **	<0.001
Resilience → PTSD	−0.21	0.10	−0.40	−0.02	−0.09 *	0.029
Resilience → Re-experiencing	−0.06	0.03	−0.11	0.00	−0.08	0.060
Resilience → Avoidance	−0.03	0.02	−0.05	−0.00	−0.09 *	0.041
Resilience → Negative alterations	−0.09	0.04	−0.16	−0.11	−0.10 *	0.020
Resilience → Hyperarousal	−0.04	0.03	−0.10	0.00	−0.06	0.197
Moderating Effects						
Smartphone addiction X Resilience → PTSD	−0.03	0.01	−0.05	−0.01	−0.09 *	0.016
Smartphone addiction X Resilience → Re-experiencing	−0.01	0.00	−0.01	0.00	−0.05	0.164
Smartphone addiction X Resilience → Avoidance	−0.01	0.00	−0.01	−0.00	−0.11 *	0.003
Smartphone addiction X Resilience → Negative alterations	−0.01	0.01	−0.02	−0.00	−0.09 **	0.010
Smartphone addiction X Resilience → Hyperarousal	−0.01	0.00	−0.01	0.00	−0.06	0.078

* *p* < 0.01, ** *p* < 0.001.

**Table 4:** The association between smartphone addiction and PTSD, avoidance, and negative alterations in mood and cognition at different levels of resilience.

Effects	B	SE	95% CI		β	*p*
			LL	UL		
Smartphone addiction → PTSD						
Low Resilience (1 *SD* below the mean)	1.28	0.14	1.00	1.56	0.46 **	<0.001
Moderate Resilience (at the mean)	1.04	0.12	0.81	1.27	0.37 **	<0.001
High Resilience (1 *SD* above the mean)	0.80	0.16	0.48	1.12	0.29 **	<0.001
Smartphone addiction → Avoidance						
Low Resilience (1 *SD* below the mean)	0.15	0.02	0.11	0.19	0.40 **	<0.001
Moderate Resilience (at the mean)	0.11	0.02	0.08	0.14	0.29 **	<0.001
High Resilience (1 *SD* above the mean)	0.07	0.02	0.02	0.11	0.18 *	0.002
Smartphone addiction → Negative alterations						
Low Resilience (1 *SD* below the mean)	0.44	0.06	0.33	0.55	0.40 **	<0.001
Moderate Resilience (at the mean)	0.34	0.05	0.25	0.43	0.31 **	<0.001
High Resilience (1 *SD* above the mean)	0.24	0.07	0.11	0.36	0.22 **	<0.001

* *p* < 0.01, ** *p* < 0.001.

**Figure A1:**
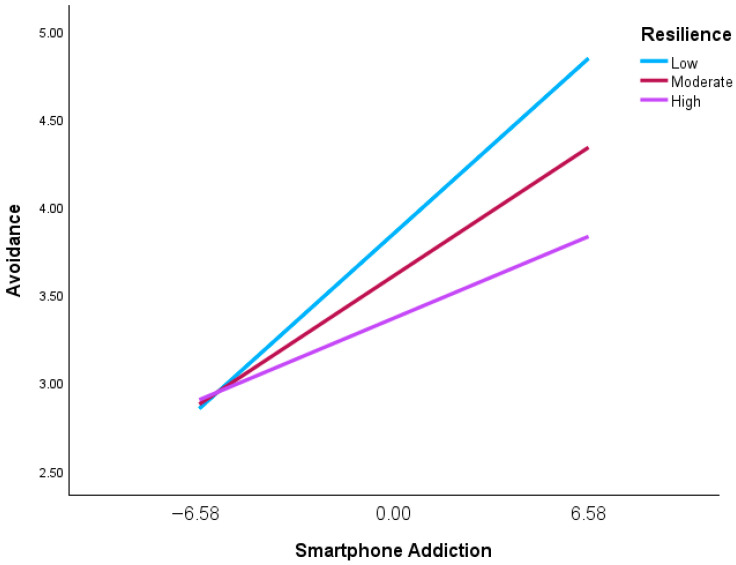
Plot of the relationship between smartphone addiction and avoidance at different values of resilience.

**Figure A2:**
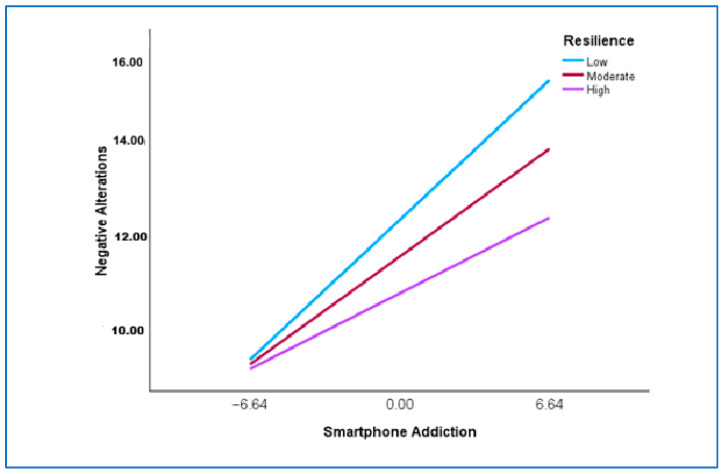
Plot of the relationship between smartphone addiction and negative alterations in mood and cognition, at different values of resilience.

## Data Availability

The raw data supporting the conclusions of this article will be made available by the authors upon request.

## Abbreviations

The following abbreviations are used in this manuscript:

SABAS	Smartphone Application-Based Addiction Scale
CDRISC-10	Connor-Davidson Resilience Scale-10
PCL-5	Posttraumatic Stress Disorder Checklist for DSM-5
PTSD	Posttraumatic Stress Disorder
